# Enoxaparin and Pentosan Polysulfate Bind to the SARS-CoV-2 Spike Protein and Human ACE2 Receptor, Inhibiting Vero Cell Infection

**DOI:** 10.3390/biomedicines10010049

**Published:** 2021-12-27

**Authors:** Maria Ennemoser, Julia Rieger, Eva Muttenthaler, Tanja Gerlza, Kurt Zatloukal, Andreas J. Kungl

**Affiliations:** 1 Department of Pharmaceutical Chemistry, Institute of Pharmaceutical Sciences, Karl-Franzens-University Graz, A-8010 Graz, Austria; maria.ennemoser@uni-graz.at (M.E.); eva.muttenthaler@edu.uni-graz.at (E.M.); tanja.gerlza@uni-graz.at (T.G.); 2 Diagnostic and Research Center for Molecular Biomedicine, Institute of Pathology, Medical University of Graz, A-8010 Graz, Austria; julia.rieger@medunigraz.at (J.R.); kurt.zatloukal@medunigraz.at (K.Z.); 3 Antagonis Biotherapeutics GmbH, Strasserhofweg 77a, A-8045 Graz, Austria

**Keywords:** SARS-CoV-2, COVID-19, Spike glycoprotein, heparan sulfate, dermatan sulfate, LMW heparin, pentosan polysulfate, PPS

## Abstract

As with many other pathogens, SARS-CoV-2 cell infection is strongly dependent on the interaction of the virus-surface Spike protein with the glycosaminoglycans of target cells. The SARS-CoV-2 Spike glycoprotein was previously shown to interact with cell-surface-exposed heparan sulfate and heparin in vitro. With the aim of using Enoxaparin as a treatment for COVID-19 patients and as prophylaxis to prevent interpersonal viral transmission, we investigated GAG binding to the Spike full-length protein, as well as to its receptor binding domain (RBD) in solution by isothermal fluorescence titration. We found that Enoxaparin bound to both protein variants with similar affinities, compared to the natural GAG ligand heparan sulfate (with Kd-values in the range of 600–680 nM). Using size-defined Enoxaparin fragments, we discovered the optimum binding for dp6 or dp8 for the full-length Spike protein, whereas the RBD did not exhibit a significant chain-length-dependent affinity for heparin oligosaccharides. The soluble ACE2 receptor was found to interact with unfractionated GAGs in the low µM Kd range, but with size-defined heparins with clearly sub-µM Kd-values. Interestingly, the structural heparin analogue, pentosan polysulfate (PPS), exhibited high binding affinities to both Spike variants as well as to the ACE2 receptor. In viral infection experiments, Enoxaparin and PPS both showed a strong inhibition of infection in a concentration range of 50–500 µg/mL. Both compounds were found to retain their inhibitory effects at 500 µg/mL in a natural biomatrix-like human sputum. Our data suggest the early topical treatment of SARS-CoV-2 infections with inhaled Enoxaparin; some clinical studies in this direction are already ongoing, and they further imply an oral or nasal prophylactic inactivation of the virus by Enoxaparin or PPS for the prevention of inter-personal viral transmission.

## 1. Introduction

As of November 2021, the ongoing COVID-19 pandemic has claimed over 5.17 million lives and severely affected our social and economic lives. Severe acute respiratory syndrome (SARS) is caused by a considerably new beta coronavirus, globally known as severe acute respiratory syndrome virus 2 (SARS-CoV-2) [1,2], and it has caused a variety of clinical morbidities and high mortality rates [3]. Clinical manifestations are versatile, ranging from asymptomatic disease progression to flu-like symptoms including fever, cough, dyspnea and fatigue, to even multiorgan failure and rapid death [4]. Although COVID-19 physiopathology is not yet completely understood, and the reasons for the strongly varying course of the disease (i.e., asymptomatic to severe disease) are still not explicitly clarified, more and more evidence assigns the disease’s severity to its corresponding levels of mild, moderate, and critical cytokine storm and the differences in individual immune responses. The so-called cytokine storm and the resulting cytokine-release syndrome (CRS) are often associated with the most severe cases [5,6]. Nevertheless, major advances were made in the comprehension of the interaction mechanism of SARS-CoV-2 and the human lung host cell [7]. A deep knowledge in this context is indispensable for designing new targeted therapies for COVID-19, and is fiercely investigated by scientists all over the world [7].

Generally, SARS-CoV-2 enters a host cell by interacting with the ACE2 receptor on the respiratory epithelial cell via its surface-anchored trimeric Spike (S) glycoprotein [8]. The SARS-CoV-2 Spike surface-protein is a trimeric 1273 amino acid glycoprotein, which can be further subdivided into S1 domain containing the receptor binding domain (RBD), including the receptor binding motif and S2 domain. Viral attachment to the ACE2 receptor is implemented through the binding of the Spike receptor binding domain to the specific, extracellular peptidase domain of ACE2 with a dissociation constant of about Kd ~ 15 nM [9]. Upon interaction, the S2 domain is exposed to proteolytic cleavage, which is crucial for subsequent viral fusion [7,10]. As already shown for other viruses, such as influenza virus, herpes simplex, and SARS-CoV-2-related viruses such as SARS-CoV and MERS-CoV, the cell-surrounding glycocalyx is often used to facilitate the binding to host cells, as this complex net of glycoproteins, fibrils and other components is in fact used by certain pathogens as co-factor for cell entry and infection [11]. Many viruses are able to interact with the negatively charged cell-surface polysaccharides, the so-called glycosaminoglycans (GAGs), among them the ubiquitously located heparan sulfate (HS). As other members of the glycosaminoglycan family, heparan sulfate can be divided in subunits of disaccharides, consisting of beta-d-glucuronic acid and alpha-d-glucosamine building blocks, which are extensively modified by various sulfations and acetylations, giving them a substantially negative charge [12]. Among all GAGs, HS exhibits the largest amount of heterogeneity, both in tissues and in an individual specific manner, as it undergoes extensive modification during and after synthesis in the Golgi apparatus [13]. In nature, HS exerts most of its functions when covalently bound to a core protein, forming the so-called HS-proteoglycans (HSPGs), which, due to their distinct GAG pattern, vary tremendously in protein-binding specificity. Additionally, their manifold biologic function, HSPGs such as the membrane-bound glypicans (GPC) and syndecans (SDC), are often used as an initial anchor point for many pathogens, including viruses, which enables the subsequent interaction of viral proteins with their receptors on the host cell, making them co-receptors for viral cell infection [3,14].

As for other viruses, also in the case of SARS-CoV-2, the binding to the ACE2 receptor is strongly dependent on the interaction between the virus-surface Spike protein and the cell-surface HSPGs [15]. The binding of the trimeric S protein to HS followed by conformational changes, enhances or even enables its binding to ACE2, rendering virus–receptor interaction and the subsequent infection more efficient [3,16]. Molecular modeling, as well as binding studies, revealed an HS binding site of the Spike glycoprotein within its S1 domain, probably in the proximity of but separate from the ACE2 binding domain of S protein, as it binds to HS as well as ACE2 in a cooperative manner [3].

Heparin represents the second member of the heparin/HS family of GAGs. Above all, it exhibits the largest overall negative charge; however, in contrast to its other family member, it plays little to no role within the biology and signaling of the extracellular matrix (ECM), as it is almost exclusively produced and stored in mast cells [17]. Heparin garnered attention due to its strong anticoagulant effects by interfering with various positions in the blood coagulation cascade. In addition, heparin possesses some anti-inflammatory, immunomodulatory, anti-viral and anti-complement activities, which may offer benefits beyond anticoagulation [18]. In contrast to unfractionated heparin (UFH), low-molecular-weight heparin (LMWH) excerpts its function only by interfering with the final common pathway of the coagulation cascade, causing the conversion of fibrinogen to fibrin through thrombin. LMW heparin blocks factor Xa and, therefore, inhibits the activation of thrombin out of prothrombin. This feature strongly reduces the risk of bleeding events in anticoagulant therapy and decreases mortality [16,19]. On a molecular level, LMW heparin is a mixture of the smaller polysaccharide fractions of the lower molecular size range, with a molecular mass of about 1.8–7.5 kDa [20]. In COVID-19, a number of studies suggests the positive effect beyond anticoagulation, as it seems to reduce disease severity and mortality [18,21,22,23,24].

**Figure 1:**
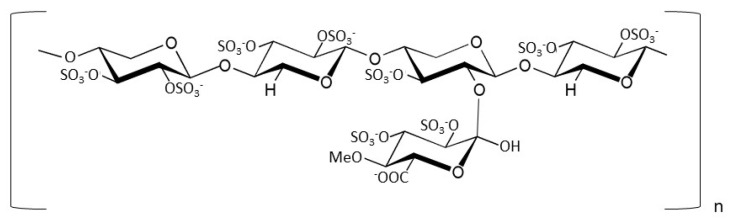
Molecular structure of pentosan polysulfate (PPS).

The heparin structural mimetic pentosan polysulfate (PPS) (Figure 1) is a semi-synthetic, polysulfated polysaccharide, naturally deriving from beechwood, with a molecular mass of 1.5–6 kDa. Due to its striking structural similarities to heparin, PPS also exhibits anticoagulant properties, albeit to a 10-fold lesser extent than its GAG counterpart. It is included in a number of pharmacological formulations, including antithrombotic prophylaxis and inflammatory conditions [25]. In the form of Elmiron^®^ capsules, it is the only FDA- and EMA-approved oral treatment for bladder discomfort associated with interstitial cystitis, as already discovered over 30 years ago [26].

At the present moment, a large number of efforts are being made to investigate the molecular and biophysical properties of Spike–GAG binding, which is shown to be crucial for infection, and to subsequently understand its potential importance in SARS-CoV-2 prophylaxis and therapy. In particular, many studies were undertaken to elucidate the role of heparin in this context, as it was proven to have an inhibitory effect on SARS-CoV-2 infection [3,16,23,27]. As already demonstrated by Hao et al. [28], HS binds to Spike and its subdomains in a sulfation degree- and position-dependent manner. When investigating heparin oligosaccharides with different sulfation patterns, it seemed that a higher degree of sulfation, especially 6O-sulfation, correlates with a higher binding affinity. In addition, the authors did not detect any influence of the chain length on heparin–ligand binding [28]. Lin Liu et al. [29] found different heparin binding affinities for the Spike proteins (1 µM for RBD, 55 nM FL), and they were able to identify HS hexa- and octasaccharides of IdoA2S-GlcNS6S as optimal ligands for Spike monomers, trimers and RBD [30]. Binding studies of the Spike protein and heparin conducted by Young et al., revealed a previously unobserved high binding affinity in the pM range [15]. After reporting a general binding of the Spike glycoprotein to heparin [16], Mycroft et al. [16] observed the significance of the 2O- and 6O-sulfation of the heparin ligand for Spike binding, as well as the minimal chain length of a hexasaccharide. In contrast, however, Kim et al. could not show a sulfation-specific dependence of the interaction, but suggested again the importance of the chain length [15].

Since all the previous studies used surface plasmon resonance methods to investigate Spike–GAG binding, for which one of the interaction partners needed to be labelled and immobilized, we performed in-solution isothermal fluorescence binding studies in order to avoid the potential influence of the surface and/or labelling on the ligand interaction. A chain length dependence of heparin binding to the Spike protein was observed but only for the full-length protein. This was not found in the viral infection inhibitory experiments, in which Enoxaparin as well as PPS were active at doses >50 µg. The binding of PPS to the Spike proteins and of GAGs, as well as of PPS to ACE2, point to a potential treatment of COVID-19 with heparin and/or PPS. Both of these molecules were found to also be virally inactivating in human sputum, which could offer the development of an oral prophylaxis of inter-personal viral transfection.

## 2. Materials and Methods

### 2.1. Recombinant Protein Production

Full-length Spike protein (FL), Spike RBD and soluble ACE2 were recombinantly expressed in Expi293 cells and were kindly provided by Ossianix Inc. (Philadelphia, PA, USA). The amino acid sequences of all proteins used in this study (including tags) are shown in Supplemental Materials.

### 2.2. Preparative Size-Exclusion Chromatography of Enoxaparin Sodium

To generate size-defined heparin compounds, preparative size-exclusion chromatography was performed according to Kitic et al. [30]. In brief, Enoxaparin sodium (Lovenox^®^, Sanofi-Aventis, Paris, France) was loaded onto a 220 cm Biogel P10 fine (Bio-Rad, Hercules, CA, USA) glass column, using 0.1 M ammonium bicarbonate buffer (Sigma Aldrich, St. Louis, MI, USA) as a mobile phase. LMW heparin was injected with a flow rate of 30 µL/min and fractions were collected every 40 min and the absorbance was recorded at 232 nm. After collecting was complete, the samples were concentrated and pooled according to the chromatogram. Concentrations were estimated using a linear heparin regression curve, measured at 232 nm. To check the purity and identity of the received dp fractions, sugar gels containing boric acid were prepared according to Gunay et al. [31] Shortly, lower chamber buffer containing 0.1 M boric acid (Sigma), 0.1 M Tris (Sigma), 0.01 M disodium EDTA (Sigma) and upper chamber buffer containing 0.2 M Tris, 1.24 M glycine (Sigma) were prepared, pH 8.4 both. An amount of 27% acrylamide (Carl Roth GmbH, Karlsruhe, Germany) gels was poured between glass plates separated by 1 mm spacers; 10 µg of each dp fraction was diluted 1:2 with lower chamber buffer containing 50% sucrose (Fluka Chemie GmbH, Seelze, Germany). Running conditions were 1 h at 160 V, followed by Azure A staining (Sigma).

### 2.3. Isothermal Fluorescence Titration

Isothermal fluorescence titration (IFT) experiments were performed using Jasco FP-6500 Spectrofluorometer at constant temperature of 20 °C, following Gerlza et al. [32]. In brief, fluorescence emission spectra of the examined proteins were recorded over the range of 300–400 nm upon excitation at 280 nm, with slit widths of 5 nm for both excitation and emission. Prior to the measurement, 100 nM protein solutions were prepared and equilibrated for 30 min. The respective GAG–ligands, LMW heparin (Lovenox^®^), HS (Celsus Laboratories Inc., Cincinnati, OH, USA), DS (Celsus), PPS, in-house produced dp4–12 and partially desulfated heparin (Iduron), were then added to final concentrations ranging from 100 nM to 3800 nM. After an equilibration time of 1 min after every GAG–ligand addition, the fluorescence spectrum was measured. For background correction, the respective GAG concentrations in PBS only were measured. The mean values of 3 independent measurements were plotted against the GAG–ligand concentration, and the resulting binding isotherm was analyzed by nonlinear regression using Origin 8.0 (Microcal Inc., Northampton, MA, USA). The dissociation constant (Kd [nM]) was calculated using following equation:

$$F=Fi+Fmax\frac{kd+\left[protein\right]+\left[ligand\right]-\sqrt{\left(Kd+\left[protein\right]+{\left[ligand\right]}^{2}\right)-4\left[protein\right]\left[ligand\right]}}{2\left[protein\right]}$$

### 2.4. Inhibition Experiments and RNA Analysis

**Figure 2:**
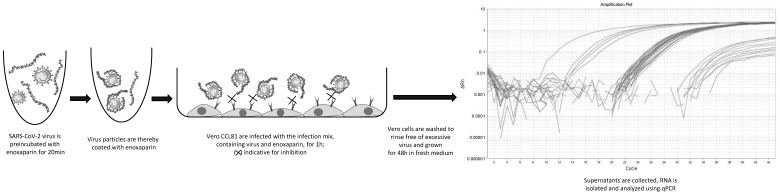
Flow chart of experimental set-up for viral inactivation experiments.

Wuhan SARS-CoV-2 (Ref-SKU: 026V-03883 Infectious cell culture supernatant of human 2019-nCoV Product Risk Group: RG3 ICTV Taxonomy: ssRNA(+)/Nidovirales/Coronaviridae/Coronavirinae/Betacoronavirus Virus name: Human 2019-nCoV ex China Strain: BavPat1/2020 Isolate: Germany ex China) strain was propagated in Vero-CCL81 cells (ATCC^®^ CCL-81™) under BSL-3 conditions. Vero-CCL81 cells were seeded in 48-well plates 24 h before infection at a cell density of 30,000 cells per well and incubated at 37 °C and 5% CO_2_ in serum free Opti Pro medium. On the day of infection, the virus (MOI 0.001) was preincubated with and without Enoxaparin and PPS for 60 min in Opti Pro medium. Vero-CCL81 cells were infected with the virus–substance mix and the virus mix alone for 1 h at 37 °C and 5% CO_2_. Non-infected cells served as negative controls and determined the background of the infection assay. After 1 h, the infection mix was removed, and cells were washed twice with phosphate-buffered saline (PBS). Following the washing procedure, fresh, pre-warmed cell culture medium was added. Samples from the supernatant were transferred to Eppendorf tubes and inactivated with 560 µL of AVL Buffer from QIAamp Viral RNA Mini Kit for subsequent RNA preparation and RT-qPCR to determine the timepoint 0 (t0) values. The virus-infected Vero-CCL81 cells were further incubated at 37 °C for 48 h. After 48 h, the supernatant was harvested and the virus was inactivated in AVL Buffer again for further quantification of SARS-CoV2 RNA by RT-qPCR (see Figure 2). Cells were visually inspected under the microscope to determine cell death due to the added substances.

To investigate these inhibitory effects on the background of a more natural biomatrix, Enoxaparin and PPS at higher concentrations were added to 250 µL sputum taken from a healthy donor. To mimic SARS-CoV-2 infection, Wuhan SARS-CoV-2 strain was added to the samples and Vero-CCL81 cells were infected as previously described. Samples from timepoint 0 h and, after 48 h incubation, were harvested in AVL Buffer again.

mRNA was isolated from supernatant at timepoint 0 h and 48 h using QIAamp Viral RNA Mini Kit according to manufacturer’s protocol. In brief, the collected and inactivated supernatant was transferred out of the BSL3 laboratory. Absolute ethanol (560 µL) was added before loading the samples completely onto the columns. Columns were washed with AW1 and AW2 buffer and the RNA was collected using 40 µL of nuclease free water (Ambion/Life Technologies, Carlsbad, CA, USA). Total isolated RNA (5 µL) was used for cDNA synthesis and qPCR, which was performed in one step using QuantiTect Probe RT-PCR (Qiagen GmbH, Hilden, Germany) on a StepOnePlus System (Applied Biosystems/Life Technologies, Carlsbad, CA, USA). CDC Primers, which were synthesized at Eurofins Scientific SE (Luxenburg), were used in a concentration of 0.4 µM and the Probe of 0.2 µM. The qPCR primers were as follows: N1 forward GAC CCC AAA ATC AGC GAA AT, N1 reverse TCT GGT TAC TGC CAG TTG AAT CTG, and N1 Probe FAM-ACC CCG CAT TAC GTT TGG TGG ACC-BHQ1. To assess RNA quality, RNase P Primers were used: RP Forward AGA TTT GGA CCT GCG AGC G, RP- Reverse GAG CGG CTG TCT CCA CAA GT and RP Probe FAM–TTC TGA CCT GAA GGC TCT GCG CG–BHQ-1. Samples were incubated at 50 °C for 30 min, heated to 95 °C for 15 min, followed by 45 cycles of 95 °C for 3 s and 55 °C for 30 s. Obtained Ct values after 48 h were subtracted from Ct values at timepoint 0 of infection and normalized between the positive control, which was set to 100% infection and untreated Vero-CCL81 cells, set to 0% infection.

## 3. Results

### 3.1. Spike FL and Spike RBD Bind GAGs and PPS with Different Affinities and Discriminate Regarding Chain-Length and Modification

First, we investigated the affinity of the Spike RBD for the naturally occurring GAGs, HS and DS, in comparison to Enoxaparin, by isothermal fluorescence titrations (Table 1). The highest affinity was detected for HS (Kd 600 ± 78.6 nM), which was slightly lower for Enoxaparin (Kd 678.4 ± 116.1 nM), but significantly lower for DS (Kd 912.5 ± 63.4 nM). Spike FL was found to bind to Enoxaparin (Kd 604.3 ± 67.4 nM) slightly better than HS (Kd 680.3 ± 66.8 nM); again, the affinity of DS was the lowest among the three GAG ligands (Kd 784.8 ± 65.6 nM). Binding studies using the natural heparin mimetic PPS revealed a high binding affinity (Kd 655 ± 118.5 nM) to Spike RBD, whereas PPS binding to Spike FL occurred with a rather low affinity (Kd 930 ± 95.7 nM).

**Figure 3:**
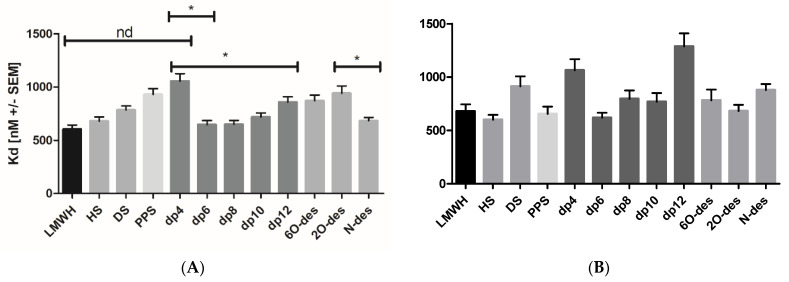
(**A**) Mean Kd values of Spike FL binding to different GAG variants with significant differences (* *p* < 0.05 was considered as statistically significant) for dp4 to dp6, dp8 dp10; dp6, dp8 to dp12; dp4 and dp12 to LMWH; LMWH to DS and PPS; LMWH to 6O-des and 2O-des; N-des to 2O-des and 6O-des; (**B**) Mean Kd values of Spike RBD binding to different GAG variants with significant differences (*) for dp4 to dp6; dp6 to dp12; dp8 and dp10 to dp12; dp12 to LMWH; HS to DS (LMWH, low molecular weight heparin = enoxaparin; HS, heparin sulfate; DS, dermatan sulfate; PPS, pentosan polysulfate; dp, depolymerisation degree; 6O-des, 6O-desulfated heparin; 2O-des, 2O-desulfated heparin; N-des, N-desulfated heparin; nd, so significance detected).

In order to investigate a potential chain-length dependence of the GAG binding affinities for the two Spike protein variants, both were subjected to IFT experiments using size-defined LMW heparin fractions ranging from dp4 to dp12. It seems that Spike FL exhibited a chain-length dependence with optimal binding to dp6 and dp8, which was less pronounced for Spike RBD (Table 1 and Figure 3). This chain-length dependence was preliminarily interpreted, as there was a minimum of one required hexa- or octasaccharide binding motif: smaller oligosaccharides cannot exert full contacts with the protein; larger oligosaccharides have too many conformational degrees of freedom which counteract high-affinity binding. In contrast to Mycroft et al. [16], who detected a binding dependent on 2O- and 6O-sulfation in SPR, our IFT interaction studies conducted with partially desulfated heparins did not provide any conclusive correlations to support their findings (Table 1). Our results were instead in agreement with the SPR results from Kim et al. [15], who found the Spike–GAG interaction to be dependent on chain length.

**Figure 4:**
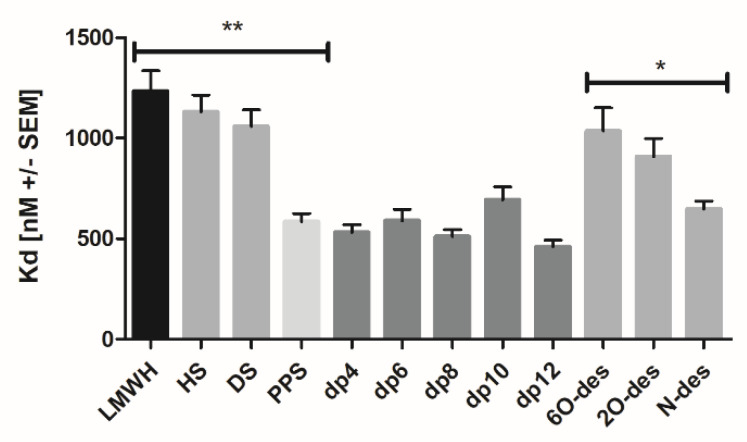
Mean Kd values of ACE2 to different GAG variants (compound abbreviations: see Figure 3; * *p* < 0.05, ** *p* < 0.01 was considered as statistically significant.

Next, we studied the GAG binding affinities of the SARS-CoV-2 protein receptor ACE2 on target cells. Interestingly, ACE2 exhibited the lowest binding to Enoxaparin (Kd 1232.58 ± 174.94 nM), and similarly higher affinities for HS and DS (Kd 1130.01 ± 145.33 nM and Kd 1057.73 ± 142.1 nM, respectively). The size-defined heparin oligosaccharides interacted with ACE2 with rather high affinities compared to the unfractionated GAGs (Table 2 and Figure 4). Additionally, the heparin mimetic PPS exhibited a lower Kd-value of 585 nM, compared to the µM affinity of the naturally occurring GAGs. Taken together, these results point to an involvement of GAG chains in the attachment of SARS-CoV-2 to cell surfaces, which could, therefore, be potentially prevented by exogenously added Enoxaparin or PPS.

### 3.2. Heparin and PPS Inhibit SARS-CoV-2 Infection In Vitro

**Figure 5:**
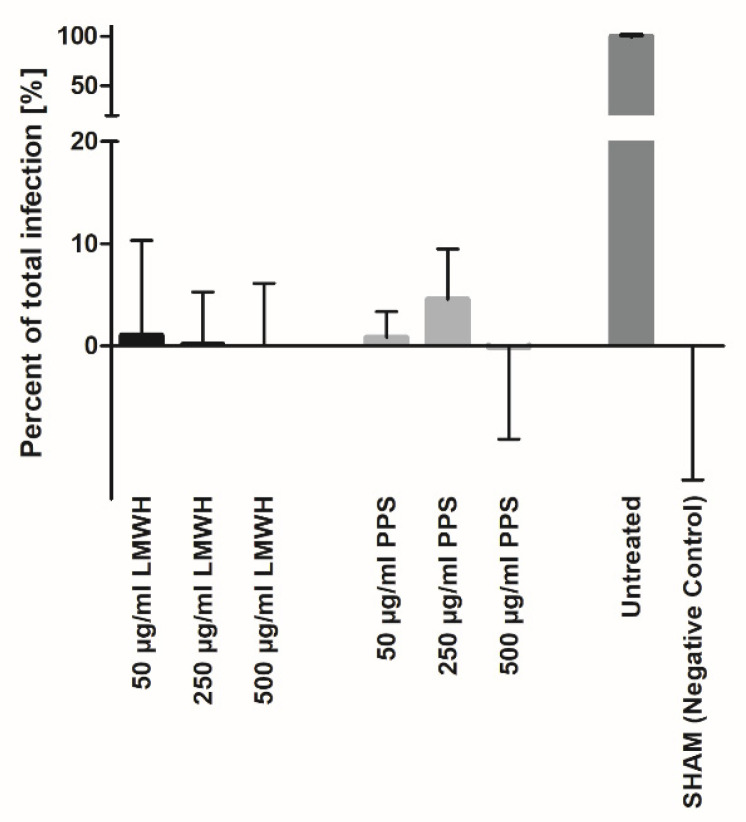
Concentration-dependent inhibition of SARS-CoV-2 cell infection of LMWH and PPS normalized to untreated positive control, as determined by RT-qPCR (SHAM: not infected, untreated cells = negative control).

To investigate the hypothesis that heparin is able to prevent the viral entry of target cells, we performed aligned infection assays with real-time PCR read-outs of viral RNA in the cell supernatant after 48 h of propagation. These inhibition experiments of viral entrance showed that Enoxaparin was able to inhibit the infection of Vero-CCL81 cells with SARS-CoV-2 in a dose-dependent manner (Figure 5). Inhibition was only achieved by incubating the virus with Enoxaparin, the pre-incubation of Vero cells with Enoxaparin did not yield inhibitory results (data not shown). Therefore, as a mode of action, we assume that Enoxaparin is able to interact with the viral Spike protein, thereby preventing interaction with its proteoglycan co-receptor on Vero cells. The Vero cells, on the other hand, cannot be targeted directly by Enoxaparin due to expected repulsive forces between proteoglycan and Enoxaparin. No significant dependence of infection inhibition efficacy on heparin chain length could be detected (data not shown). Our results are in accordance with recently published data [3,16,27]. Interestingly, the heparin mimetic PPS showed a similar inhibition efficacy of viral infection to Enoxaparin (Figure 5), which relates to the structural similarity of the two compounds.

### 3.3. Heparin and PPS Inhibit SARS-CoV-2 Infection in a Biomatrix-like Environment

**Figure 6:**
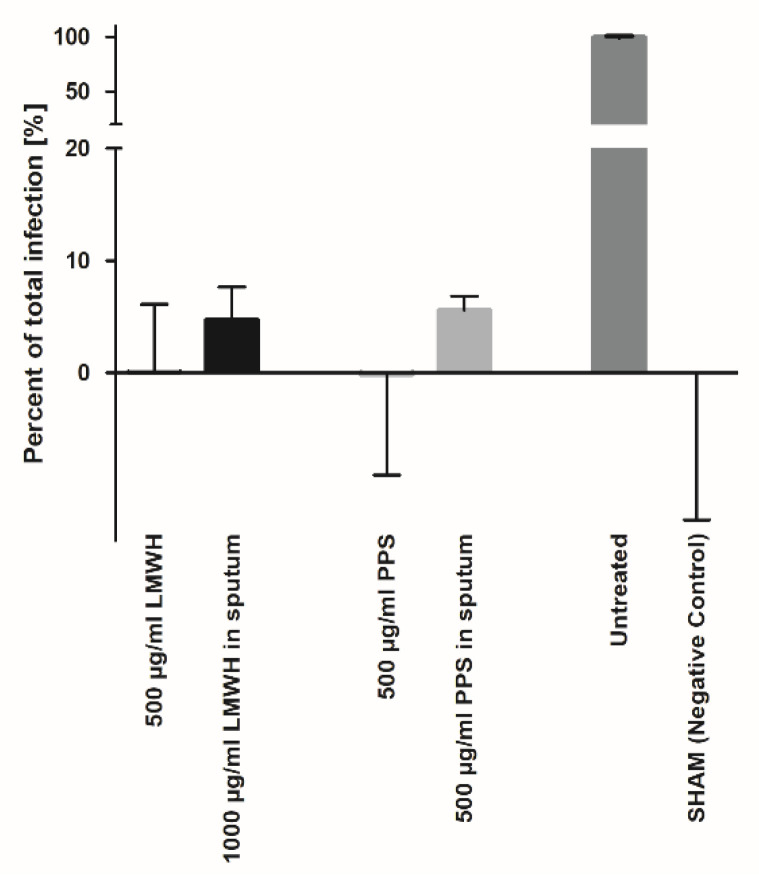
Inhibition of cell infection by LMWH and PPS in cell culture media and in human sputum sample samples.

Based on our results, a potential indication of Enoxaparin could be envisaged as an oral prophylaxis of viral transmission. For this purpose, the route of application would be via human sputum, e.g., in the form of Enoxaparin-containing lozenges. Therefore, we performed SARS-CoV-2 infection inhibition experiments on Vero-CCL81 cells in sputum collected from healthy individuals. Due to the complex biomatrix containing a number of GAG-binding proteins (the results of a comprehensive GAG-binding proteome study of sputum are currently prepared for publication; Almer et al.), the Enoxaparin dose had to be increased to 1 mg/mL in order to achieve an inhibition efficacy similar to the efficiency in the cell culture medium (Figure 6). Interestingly, PPS exhibited a strong inhibition efficiency already at a concentration of 500 µg/mL, similar to the efficiency obtained in the cell culture medium.

## 4. Discussion

The most abundant GAG in the ECM of the human lung, aside from CS/DS, is HS [33]. A large number of previous studies already identified HS as a crucial co-factor in the SARS-CoV-2 infection cascade and characterized the binding of HS to the Spike glycoprotein of SARS-CoV-2 [1,3]. Within our experimental series we were able to successfully compare binding affinities of the viral Spike glycoprotein with HS as well as with CS. Unlike in previous studies, we investigated these interactions in a solution, thereby avoiding immobilizing one of the interaction partners. Our experiments highlighted HS as a better binder compared with CS binding to both Spike FL and RBD. In binding studies, using LMW heparin (Enoxaparin) as a GAG ligand gave affinities similar to HS, suggesting Enoxaparin as a potential competitor of the HS-supported viral infection of target cells.

Since typical Enoxaparin preparations consist of a mixture of heparin molecules with different molecular sizes, we further sub-fractionated the Enoxaparin sample by means of SEC. The resulting oligosaccharides were analyzed with respect to a potential optimum binding chain length. Our binding studies showed the best binding for the medium-sized fractions of LMW heparin, presenting dp4 and dp12 as less potent in Spike binding. This effect of size discrimination was more pronounced in full-length Spike and not so much in RBD (Figure 3). In addition to the size dependence of the Enoxaparin–Spike interactions, the influence of particular Enoxaparin sulfation sites on Spike affinities was investigated (Table 1 and Figure 3). Again, full-length Spike proteins exhibited a certain potency in discriminating between partially desulfated heparins revealing N-sulfation sites as inhibiting high-affinity Enoxaparin binding. A selective binding of heparin to the spike protein can also be deduced from recently published modeled complex structures [34,35].

Taken together, these results, especially the HS-like binding affinity of LMW heparin to the Spike protein, suggest a potential competition between endogenous HS, as a co-receptor of the virus, and exogenously added heparin. Additionally, although heparin binding to the RBD of Spike was observed, the full-length Spike protein seems to be required to differentiate between different-sized and partially desulfated heparins, indicating two GAG binding sites on the Spike protein.

Considering the eminent binding of GAGs to the viral Spike protein, we were interested to find out if the ACE2 receptor would also bind to GAGs, thereby implying a triple complex consisting of Spike, ACE2 and GAG/HS to be the active form of the viral docking complex. Compared to the Spike protein, ACE2 exhibited an overall lower affinity for the three GAG classes investigated here (Table 2 and Figure 4). Only PPS was found to interact with ACE2, with a high binding affinity of 585 nM. Interestingly, all size-defined heparin oligosaccharides bound to ACE2 with a much higher affinity than the unfractionated Enoxaparin. This is indicative for a narrow GAG binding site on ACE2. The affinity of ACE2 for heparin was found to also be dependent upon the presence of certain sulfation, with Kd values decreasing significantly after 6O- < 2O- < N-desulfation. In short, N-desulfated GAG sections could, therefore, constitute the binding interface between HS and ACE2, which is indicative for flexible, largely desulfated loops of HS.

The preincubation of SARS-CoV-2 virus with LMW heparin (Enoxaparin) led to a concentration-dependent inhibition of SARS-CoV-2 infection of Vero-CCL81 target cells (Figure 5). This is interpreted as a coating of the virus by Enoxaparin, which subsequently inhibits binding to the HS co-receptor(s) on the target cell surface, thus preventing infection. Additionally, although pre-incubation of the target cells with Enoxaparin, enabling potential ACE2 interactions, did not lead to a similar inhibition of viral entry/propagation in the target cells (data not shown), it can be assumed that a Spike/Enoxaparin complex would be unable to crack a pre-formed complex of ACE2 and cell-surface GAG/HS. It can thus be hypothesized that Enoxaparin not only prevents SARS-CoV-2 infection via blocking its HS co-receptor on target cells, but also impacts the binding of Spike to its primary receptor ACE2.

Pentosan polysulfate (PPS) is a natural GAG/heparin molecular mimetic derived from beech trees, which is in clinical use, but not as a blood anti-coagulant. Since we intend to profile the Spike/ACE2/GAG axis as a novel therapeutic interface for the treatment and prevention of SARS-CoV-2 virus transmission, a compound with less potential side effects (e.g., bleeding) than heparin was investigated. Interestingly, the binding of PPS to Spike was found to be significant but less efficacious than binding to ACE2. In viral infection experiments, PPS inhibited entry/propagation of the virus in a similar concentration-dependent manner as Enoxaparin. We can therefore assume, that both, Enoxaparin and PPS, are potent anti-COVID-19 compounds which prevent viral spread in vitro, and are highly efficacious at low active doses (Figure 5).

As mentioned above, a very straightforward way to make use of the anti-viral activity of Enoxaparin and PPS would be to apply the compounds orally (or nasally) at low doses, thereby neutralizing the virus in the sputum (nasal fluid) which would prevent oral/nasal transmission of the virus very efficiently. We therefore tested the neutralizing ability of Enoxaparin and PPS in sputum samples of healthy donors (Figure 6). Additionally, although the activity of the compounds to reduce viral spread is slightly reduced in sputum compared to PBS, the doses required to disable the virus in sputum are still in a very attractive range (500 µg/mL), allowing them to be formulated as a lozenge or a nebulizer for oral and/or nasal applications.

Our data show that enoxaparin is a very effective inhibitor of SARS-CoV-2 infection and/or of propagation of the virus. At the low doses applied, the compound is not expected to induce strong side effects, especially since it was recently shown that high oral doses of heparin, with the aim of systemic availability, did not show noticeable off-target reactions [36]. We can, therefore, expect a strong reduction in the active viral load in the oral cavity (and in the nose if applied as respective spray) of a SARS-CoV-2 carrier, which leads to a significant reduction in infectious viral transmission by the carrier. A direct therapeutic effect on the COVID-19 carrier, however, is not expected since the oral bioavailability of heparin is very poor [36]. Aside from this, a novel use (a re-purposing) of enoxaparin as a viral-inactivating compound, formulated as a mouth/nose spray or as lozenge, is strongly suggested by our results.

## 5. Conclusions

We presented evidence that enoxaparin interacts with the spike protein of SARS-CoV-2 virus, thereby preventing viral infection/propagation in target cells. The inhibitory effect of heparin was, moreover, found to be dependent upon the chain length and on the sulfation pattern present on the heparin molecules. A heparin structural analogue, PPS, showed a similar inhibitory activity of viral infection with a differentiated binding profile to the ACE2 receptor compared to enoxaparin. Since both compounds, enoxaparin and PPS, were found to be inhibitory of viral infection/propagation in human sputum, they are proposed to be applicable in a therapeutic, inhaled setting, as well as in an anti-transmission setup as a spray or as a lozenge. All routes of application are currently under investigation for clinical trials, with a prioritization given to the prevention of active virus aerosolization and transmission by a mouth rinse with low doses of enoxaparin. If the clinical trials lead to the expected results, i.e., a strong reduction in the active/infective viral load in the mouth (and nose) of a COVID-19-positive individual, we expect a strong social impact of our approach on interpersonal contacts in cases where potential viral bearers cannot be identified since SARS-CoV-2 tests cannot be performed easily and with the required high frequency (e.g., at dentists, schools, etc.).

## 6. Patents

A European priority patent submission with the title, “Novel use of heparin and heparin analogues” (EP 21155990.1), has been filed.

## Figures and Tables

**Table 1:** Kd values (nM) for Spike FL and Spike RBD to different GAG variants.

	Spike FL	Spike RBD
Enoxaparin	604.3 ± 67.4	678.4 ± 116.1
HS	680.3 ± 66.8	600 ± 78.6
DS	784.8 ± 65.6	912.5 ± 163.4
PSS	930 ± 95.7	655 ± 118.5
Dp4	1054.7 ± 122.9	1063.3 ± 180.1
Dp6	646.6 ± 65.2	619.2 ± 80.4
Dp8	648.8 ± 64.6	795.6 ± 137.3
Dp10	718.5 ± 66.6	768.7 ± 140.7
Dp12	857.9 ± 88.1	1287 ± 213
6O-des	870.3 ± 92.2	781.4 ± 175.7
2O-des	940.2 ± 121.7	681.9 ± 175.7
N-des	682.2 ± 55.9	878.5 ± 96.4

**Table 2:** Kd values (nM) for ACE2 to HS, DS, Enoxaparin, PPS and size-defined LMWH fractions.

	ACE2
Enoxaparin	1232.6 ± 174.9
HS	1130 ± 145.3
DS	1057.7 ± 142.1
PSS	585 ± 41
Dp4	531.6 ± 38.6
Dp6	590.1 ± 56
Dp8	509.9 ± 35
Dp10	692.9 ± 65
Dp12	460.7 ± 33
6O-des	1034 ± 116
2O-des	908 ± 90
N-des	647 ± 40

## Data Availability

Additional sequence data can be found in Supplementary Material.

## Supplementary Materials

The following are available online at https://www.mdpi.com/article/10.3390/biomedicines10010049/s1.
