# Supplementary material for: Enoxaparin and Pentosan Polysulfate Bind to the SARS-CoV-2 Spike Protein and Human ACE2 Receptor, Inhibiting Vero Cell Infection
Source: Biomedicines. 2021 Dec 27;10(1):49. doi: 10.3390/biomedicines10010049 (PMC8772983; doi:10.3390/biomedicines10010049)
Supplement: Supplementary file 1 [file biomedicines-10-00049-s001.zip › biomedicines-1510490-supplementary.pdf]

SUPPLEMENTAL MATERIAL

(Ennemoser et al._Biomedicines 1510490, Revision)

Spike RBD (S1-RBD 319-541)

```
        10          20          30          40          50          60
RVQPTESIVR  FPNITNLCPF  GEVFNATRFA  SVYAWNRKRI  SNCVADYSVL  YNSASFSTFK

        70          80          90         100         110         120
CYGVSPTKLN  DLCFTNVYAD  SFVIRGDEVR  QIAPGQTGKI  ADYNYKLPDD  FTGCVIAWNS

       130         140         150         160         170         180
NNLDSKVGGN  YNYLYRLFRK  SNLKPFERDI  STEIYQAGST  PCNGVEGFNC  YFPLQSYGFQ

       190                200                 210                 220
PTNGVGYQPY RVVVLSFELL HAPATVCGPK KSTNLVKNKC VNF
```

Tag: AVI-Tag mit 6xHis separated by G_4S linker

Spike FL (S1 subdomain 16-685)

```
        10          20          30          40          50          60
VNLTTRTQLP  PAYTNSFTRG  VYYPDKVFRS  SVLHSTQDLF  LPFFSNVTWF  HAIHVSGTNG

        70          80          90         100         110         120
TKRFDNPVLP  FNDGVYFAST  EKSNIIRGWI  FGTTLDSKTQ  SLLIVNNATN  VVIKVCEFQF

       130         140         150         160         170         180
CNDPFLGVYY  HKNNKSWMES  EFRVYSSANN  CTFEYVSQPF  LMDLEGKQGN  FKNLREFVFK

       190         200         210         220         230         240
NIDGYFKIYS  KHTPINLVRD  LPQGFSALEP  LVDLPIGINI  TRFQTLLALH  RSYLTPGDSS

       250         260         270         280         290         300
SGWTAGAAAY  YVGYLQPRTF  LLKYNENGTI  TDAVDCALDP  LSETKCTLKS  FTVEKGIYQT

       310         320         330         340         350         360
SNFRVQPTES  IVRFPNITNL  CPFGEVFNAT  RFASVYAWNR  KRISNCVADY  SVLYNSASFS

       370         380         390         400         410         420
TFKCYGVSPT  KLNDLCFTNV  YADSFVIRGD  EVRQIAPGQT  GKIADYNYKL  PDDFTGCVIA

       430         440         450         460         470         480
WNSNNLDSKV  GGNYNYLYRL  FRKSNLKPFE  RDISTEIYQA  GSTPCNGVEG  FNCYFPLQSY

       490         500         510         520         530         540
GFQPTNGVGY  QPYRVVVLSF  ELLHAPATVC  GPKKSTNLVK  NKCVNFNFNG  LTGTGVLTES

       550         560         570         580         590         600
NKKFLPFQQF  GRDIADTTDA  VRDPQTLEIL  DITPCSFGGV  SVITPGTNTS  NQVAVLYQDV

       610              620               630               640               650
NCTEVPVAIH ADQLTPTWRV YSTGSNVFQT RAGCLIGAEH VNNSYECDIP IGAGICA
```

Tag: AVI-Tag with 6xHis separated by G_4S linker

ACE2 (ectodomain 18-740)

```
        10          20          30          40          50          60
QSTIEEQAKT  FLDKFNHEAE  DLFYQSSLAS  WNYNTNITEE  NVQNMNNAGD  KWSAFLKEQS

        70          80          90         100         110         120
```

```
TLAQMYPLQE   IQNLTVKLQL   QALQQNGSSV   LSEDKSKRLN   TILNTMSTIY   STGKVCNPDN

       130          140          150          160          170          180
PQECLLLEPG   LNEIMANSLD   YNERLWAWES   WRSEVGKQLR   PLYEEYVVLK   NEMARANHYE

       190          200          210          220          230          240
DYGDYWRGDY   EVNGVDGYDY   SRGQLIEDVE   HTFEEIKPLY   EHLHAYVRAK   LMNAYPSYIS

       250          260          270          280          290          300
PIGCLPAHLL   GDMWGRFWTN   LYSLTVPFGQ   KPNIDVTDAM   VDQAWDAQRI   FKEAEKFFVS

       310          320          330          340          350          360
VGLPNMTQGF   WENSMLTDPG   NVQKAVCHPT   AWDLGKGDFR   ILMCTKVTMD   DFLTAHHEMG

       370          380          390          400          410          420
HIQYDMAYAA   QPFLLRNGAN   EGFHEAVGEI   MSLSAATPKH   LKSIGLLSPD   FQEDNETEIN

       430          440          450          460          470          480
FLLKQALTIV   GTLPFTYMLE   KWRWMVFKGE   IPKDQWMKKW   WEMKREIVGV   VEPVPHDETY

       490          500          510          520          530          540
CDPASLFHVS   NDYSFIRYYT   RTLYQFQFQE   ALCQAAKHEG   PLHKCDISNS   TEAGQKLFNM

       550          560          570          580          590          600
LRLGKSEPWT   LALENVVGAK   NMNVRPLLNY   FEPLFTWLKD   QNKNSFVGWS   TDWSPYADQS

       610          620          630          640          650          660
IKVRISLKSA   LGDKAYEWND   NEMYLFRSSV   AYAMRQYFLK   VKNQMILFGE   EDVRVANLKP

       670          680          690          700          710          720
RISFNFFVTA PKNVSDIIPR TEVEKAIRMS RSRINDAFRL NDNSLEFLGI QPTLGPPN    QPPVS
```

Tag: FLAG with 6xHis separated by G_4S
